# A comparison of explainable artificial intelligence methods in the phase classification of multi-principal element alloys

**DOI:** 10.1038/s41598-022-15618-4

**Published:** 2022-07-08

**Authors:** Kyungtae Lee, Mukil V. Ayyasamy, Yangfeng Ji, Prasanna V. Balachandran

**Affiliations:** 1 Department of Materials Science and Engineering, University of Virginia, Charlottesville, VA 22904 USA; 2 Department of Computer Science, University of Virginia, Charlottesville, VA 22904 USA; 3 Department of Mechanical and Aerospace Engineering, University of Virginia, Charlottesville, VA 22904 USA

**Keywords:** Structural materials, Metals and alloys, Materials science, Theory and computation, Coarse-grained models

## Abstract

We demonstrate the capabilities of two model-agnostic local post-hoc model interpretability methods, namely breakDown (BD) and shapley (SHAP), to explain the predictions of a black-box classification learning model that establishes a quantitative relationship between chemical composition and multi-principal element alloys (MPEA) phase formation. We trained an ensemble of support vector machines using a dataset with 1,821 instances, 12 features with low pair-wise correlation, and seven phase labels. Feature contributions to the model prediction are computed by BD and SHAP for each composition. The resulting BD and SHAP transformed data are then used as inputs to identify similar composition groups using *k*-means clustering. Explanation-of-clusters by features reveal that the results from SHAP agree more closely with the literature. Visualization of compositions within a cluster using Ceteris-Paribus (CP) profile plots show the functional dependencies between the feature values and predicted response. Despite the differences between BD and SHAP in variable attribution, only minor changes were observed in the CP profile plots. Explanation-of-clusters by examples show that the clusters that share a common phase label contain similar compositions, which clarifies the similar-looking CP profile trends. Two plausible reasons are identified to describe this observation: (1) In the limits of a dataset with independent and non-interacting features, BD and SHAP show promise in recognizing MPEA composition clusters with similar phase labels. (2) There is more than one explanation for the MPEA phase formation rules with respect to the set of features considered in this work.

## Introduction

Machine learning (ML) techniques have been actively utilized for various applications in recent years due to their superior prediction performance. However, as the applications expand vastly, they also have been required to meet the demand of high level of transparency and accountability due to their black-box framework^1–9^. As a result, explainable ML methods have attracted a great deal of attention in order to further advance the reliability and applicability of ML-based approaches. Model explainability is also becoming increasingly important in the materials science domain as ML and artificial intelligence (AI)-driven algorithms are beginning to show success in simplifying various workflows^10–22^. However, the idea of incorporating explainable ML methods into the current data-driven materials design and discovery workflow is still in its infancy. Unlike many other disciplines, materials science suffers from limited and sparse data. Incorporating interpretablility into the black-box ML models make it possible to explain the ML predictions with more confidence, especially when the models are trained using small and heterogeneous datasets. Explainable ML methods improve the trustworthiness of the trained models by uncovering the biases and errors inside of the models, which would not be identified otherwise using standard goodness-of-fit metrics such as R$^2$, mean squared error, mean absolute error, precision, and recall to name a few. Overall, the interpretable and explainable ML framework makes the ML paradigm more accessible to domain experts.

One of the representative and popular methods for model explanations is SHapley Additive exPlanations (SHAP) developed by Lundberg and Lee^23^. SHAP is a post-hoc, local model-agnostic explanation method based on the SHAPley values that were introduced in cooperative game theory to calculate the contributions of players to the total payout^24^. In predictive models, the contribution of each feature can be calculated by averaging over every possible ordering of features using SHAP, allowing one to locally analyze the importance of each input feature for a given instance prediction. Thus, the SHAP method calculates each feature contribution by evaluating a change in the expected model prediction when conditioned on a given feature. In the SHAP algorithm, each feature is assigned an importance value that signifies its effect on the model prediction as a result of their inclusion in the model. To calculate the importance values, a model is trained in the presence of each corresponding feature, while another model is also trained with the feature values withheld. Finally, the predictions from those two models are compared and the SHAP values are computed by averaging the differences for all possible subsets as the effect of withholding a feature is influenced by other features in the model^24^. SHAP can be used to provide both local and global understanding of a trained ML model. For these reasons, the SHAP method has been the predominant method used to analyze machine learning results in various materials science publications on alloys, catalysts, photovoltaics, metal-organic frameworks and oxide glasses to name a few^13,25–29^.

In this paper, in addition to SHAP, we will focus on two other instance-level post-hoc model interpretability approaches, namely breakDown (BD) analysis and Ceteris-Paribus (CP) profiles, that have received little attention in the materials science community. The BD method, similar to the SHAP method, is also based on the variable attribution principle that decomposes the prediction of each individual observation into particular variable contributions^30,31^. Unlike the SHAP values, the BD values provide order specific explanations of variables’ contributions in a greedy way^5^. One of the assumptions of BD method is that the input features (variables or descriptors) are independent and non-interacting^32^. There are two algorithms for BD analysis: (1) step-down and (2) step-up. The step-down method starts from a full set of input features. Then, each individual feature contribution is calculated by sequentially removing a single feature from a set followed by variable relaxation in order that the distance to the model prediction is minimized. The step-up method starts from a null set and follows the opposite direction of the step-down method. Both methods have been shown to provide consistent outcomes in feature contributions. At a cursory glance, these assumptions and definitions appear to suggest that the BD method for post-hoc model interpretability may find a natural home in explaining hierarchical learning algorithms that preserve a taxonomy. However, Gosiewska and Biecek^33^ argue that complex predictive models are usually non-additive and model interpretation should depend on the order in which the explanation is read. Therefore, setting a proper visit order may lead to a better and intuitive understanding of the model prediction. There are papers in the published literature, where the BD method is used for post-hoc model interpretability in a non-hierarchical learning setting^5,34–36^. In this paper, we use the BD data from our recent published work^37^, where the step-down method was used for variable attribution.

On the other hand, the CP profiles, also referred to as individual conditional expectations (ICE) plots, evaluate the influence of a variable from a trained ML model under the assumption that the values of all other variables are fixed (akin to what-if analysis)^38^. The dependence between the predicted response and a feature is visualized by CP profiles, where one can observe how the input and responses are related at a glance (e.g. in a linear, non-linear or more complex pattern). In this way, the CP analysis helps to quantify the impact of a given variable on the predictions of a black-box model and provide a cursory, visual explanation of the functional form connecting an input with the output. Insights about such a functional dependence is not readily ascertained from either the SHAP or BD analysis. As a result, there is immense value in complementing SHAP and BD analyses with CP profile plots. In the Methods section, we provide a brief mathematical background of SHAP, BD, and CP profiles methods.

Although the BD, SHAP and CP profile plots are representative methods for post-hoc model interpretation, the comparison of these methods have been rarely reported in ML literature. Lorentzen and Mayer analyzed the policies of motor third-party liability and compared the SHAP decomposition with the BD method^34^. However, the differences were not discussed in sufficient detail. Rinzivillo et al. also reported both SHAP and BD decompositions, but made a simple comparison on the feature importance plots from these methods without a further analysis about the differences^35^. Gosiewska and Biecek examined the dataset about the sinking of the Titanic using BD and SHAP. They presented a different aspect of feature importance from each method, mentioning that it was not clear to determine which one is more reliable. Their local interpretability was modified by including the interaction factors between descriptors, but no comparison between BD and SHAP was made thereafter^33^. Thus, a comparative study on BD and SHAP is important to better understand the usage and effectiveness of these methods. In term of run time and computational complexity, the BD algorithm runs faster than the SHAP algorithm^32,39^.

In this paper, we present a comparative study of two post-hoc model interpretability methods using the phase classification problem of multi-principal element alloys (MPEAs). The MPEAs make a good template for this study because the dataset is high-dimensional and has 1,821 compositions in seven class labels (additional details given in the Methods Section). The MPEA phase classification problem is well-studied in the literature^40–45^, which provides us with sufficient domain knowledge to compare the outcomes from SHAP and BD results. Previously, we reported a ML study result about BD analysis based an ensemble of support vector machine (eSVM) along with the *k*-means clustering method, followed by CP analysis^37^. The objective of this paper is three-fold: (1) Demonstrate that the post-hoc model interpretability methods can identify complex patterns in high-dimensional data sets and identify data points with similar phase labels (compared to the raw data). (2) Uncover factors that have led to the aggregation of data points with similar phase labels. Compare two complementary post-hoc model interpretability methods, namely BD and SHAP, by identifying the similarities and differences between the two methods. (3) Extract insights into phase formation rules of MPEAs. Specifically, the local feature importance weights for each instance are separately computed by the BD and SHAP methods. The resulting two different sets of variable attribution data are then independently clustered by the *k*-means clustering algorithm. Both BD and SHAP approaches turn out to be successful in capturing the similarities between the compositions that represent the various MPEA phase labels in the dataset. Explanation-of-clusters by features reveal commonalities and differences between BD and SHAP methods. Although the SHAP results are found to be more consistent with the literature, both BD and SHAP values capture well the known findings in the published literature. Despite approaching local feature importance from different perspectives and assumptions, explanation-of-clusters by examples reveal grouping of similar compositions within the clusters. A detailed analysis of the CP profile plots for the compositions within the clusters show similar functional dependencies between the feature values and predicted responses. Interpretation of the results provide insights into the phase formation rules of MPEAs.

## Results

**Figure 1:**
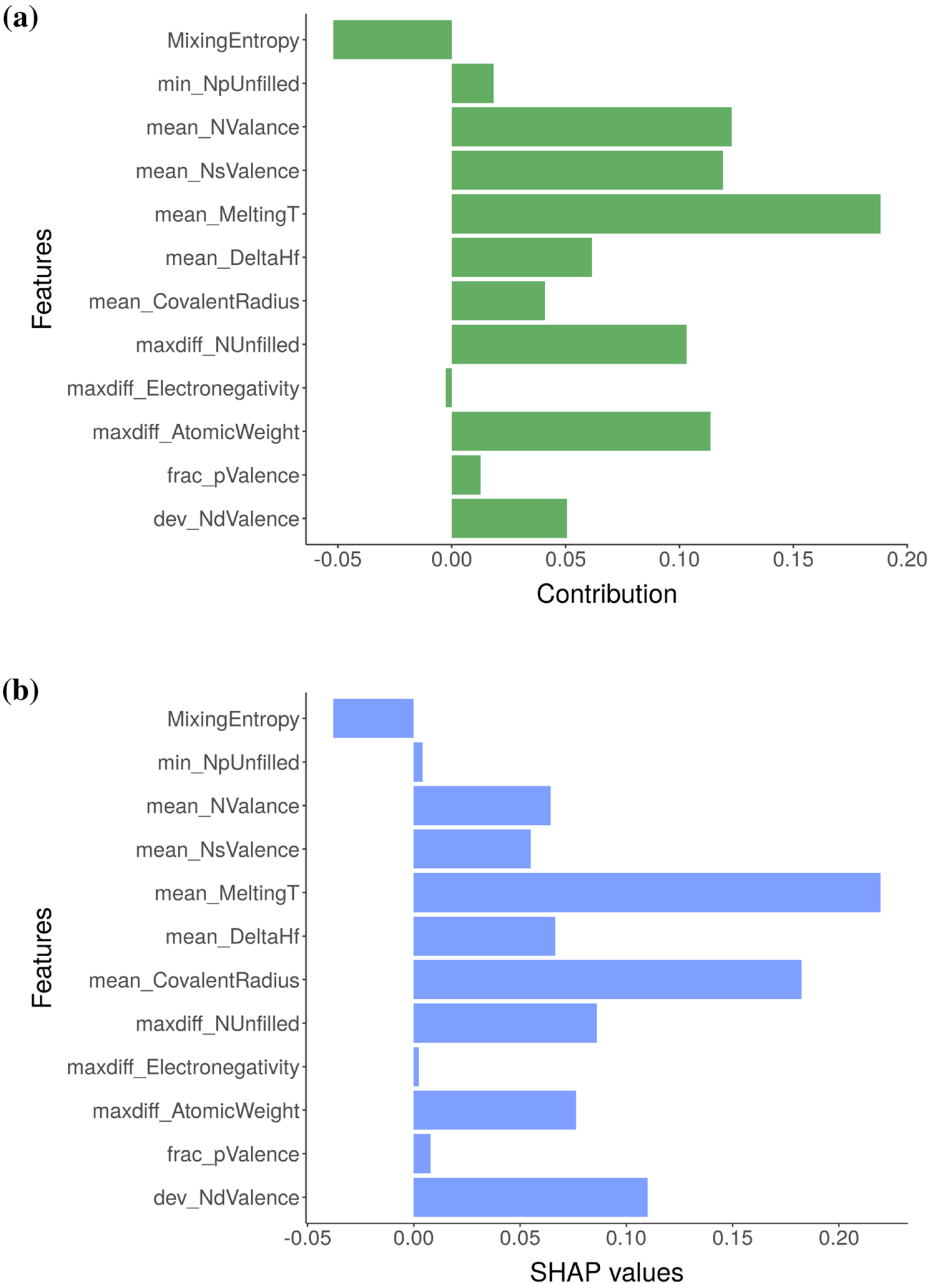
The (**a**) BD and (**b**) SHAP decomposition are plotted for the NbTaTiV composition, which is predicted to form in the BCC phase by the eSVM model. The y-axis of SHAP plot is sorted in the same way as that of the BD plot to facilitate the comparison of two plots.

In Fig. 1, we discuss the explanation-by-example using the NbTaTiV composition as a template, which is predicted to form in BCC structure by our eSVM model. Both BD contributions and SHAP values are expressed as bar graphs, where positive and negative values indicate the contribution of each variable to the overall prediction. Both plots show the relative importance of mean_meltingT to explain the prediction of the NbTaTiV composition. Some differences in the BD and SHAP variable attributions can also be seen. The dev_NdValence and mean_ConvalentRadius are identified as important variables by SHAP, whereas the BD analysis identifies mean_NValence and mean_NsValence as important. Our key point from Fig. 1 is the following: even in this simple case, it is important to note that the SHAP and BD results do not completely agree with each other. This is not entirely surprising because in SHAP the contribution of a feature is averaged over all possible conditional expectations, whereas in the greedy BD method only a single order (Fig. 1a) of conditional expectation is performed^5^. In a similar vein, we have calculated the BD and SHAP values for every alloy composition in the training data.

In order to better understand the differences between the BD and SHAP methods, we now transition to model explainability at an intermediate level, where the instances are clustered based on similarities of their attributes as represented by the BD and SHAP values. Although the variable attribution from SHAP and BD for an individual instance may appear differently, does this mean they capture vastly different pattern in the data? The main purpose is to understand and explain similar compositions within a cluster. From the materials science standpoint, clusters are expected to shed insights into the key factors that govern the formation of a particular phase as a function of chemical composition. As a result, there is value in such analysis.

**Figure 2:**
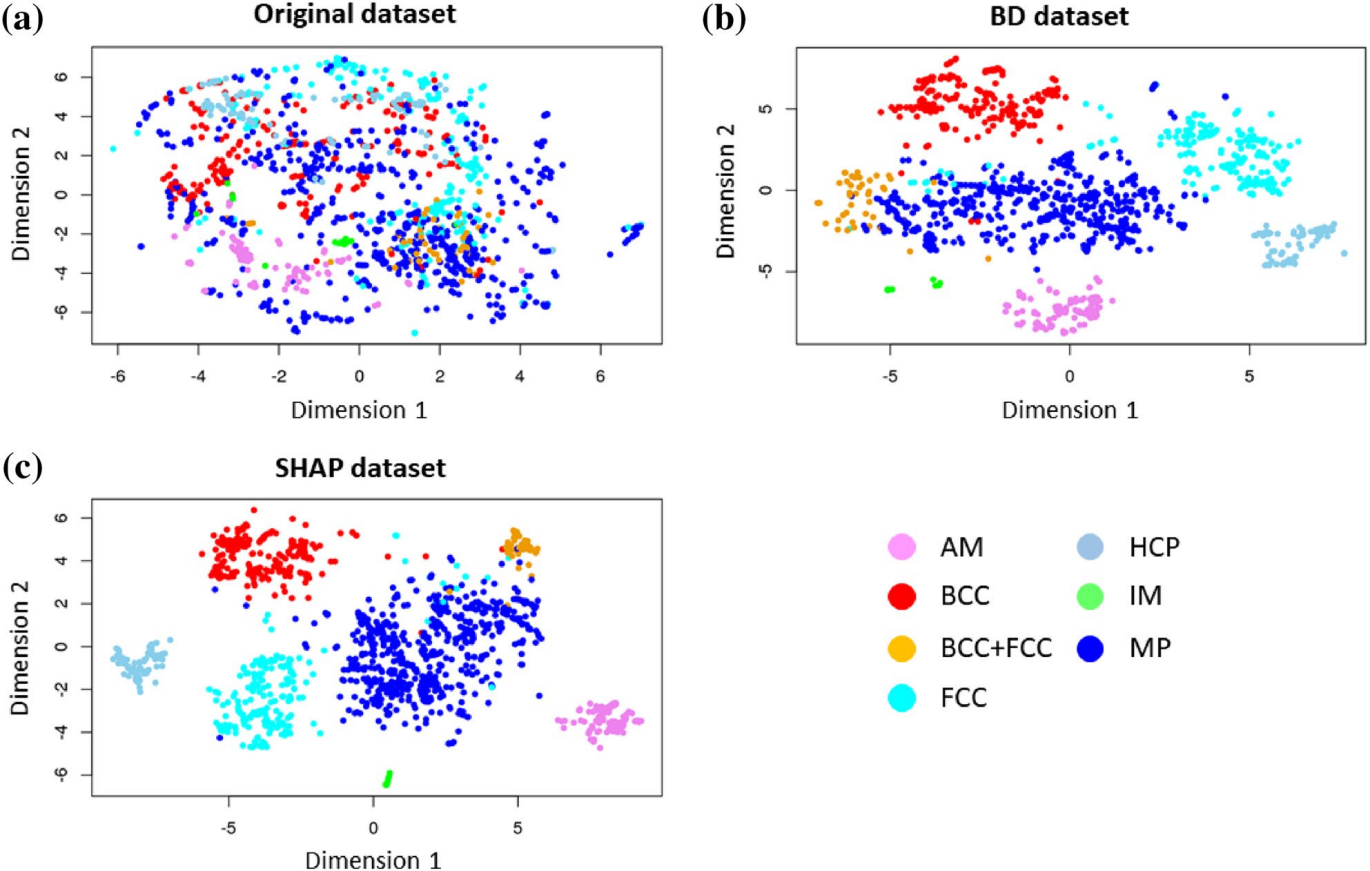
Three different high-dimensional datasets visualized using the t-SNE algorithm: (**a**) original or raw data, (**b**) BD, and (**c**) SHAP.

Figure 2 shows two-dimensional projections of high-dimensional datasets based on the t-distributed stochastic neighborhood embedding (t-SNE) method^46^. Compared to the raw or original data set (Fig. 2a), the BD (Fig. 2b) and SHAP (Fig. 2c) data sets capture aggregation of chemical compositions with similar phase labels. Although this result is expected because the BD and SHAP methods exhibit the property of local accuracy (see Methods section), the plots shown in Fig. 2b and c indicate that the BD and SHAP data sets carry information to extract insights into the various phase labels in this MPEA data set.

In our earlier work^37^, we introduced a novel algorithm (see Supplementary Algorithm S1) that integrated BD values, *k*-means clustering and CP profile plots based on the eSVM framework. We briefly discuss the significance of this algorithm. We have a high-dimensional input feature space and when we visualized it using the t-SNE method we found that the MPEA phases are non-separable (Fig. 2a). The features in the input space have low pair-wise correlation coefficient (see Methods), thus the independence assumption is weakly satisfied. We also do not explicitly code feature interactions in the input space. The supervised eSVM method with radial-basis kernel function generates a non-linear decision boundary that led to a classification accuracy of 86% on the test data (see Methods). The next step involves performing post-hoc model interpretability analysis of the trained eSVM model based on the BD method. We interpret that the BD analysis reformulates the non-linear eSVM mapping into an approximated linear space by using local accuracy approximation (see Methods section), where we have a variable importance or weight associated with each input feature. We then redefine every composition (or instance) in our dataset in terms of the variable importance as calculated from the BD analysis. The redefined dataset serves as an input for *k*-means clustering, which in turn identifies similar composition groups. We then visualized the clusters by CP profile plots to explain the similarities and differences. Thus our algorithm provides a unique way to understand the outcomes from the post-hoc interpretability methods.

**Figure 3:**
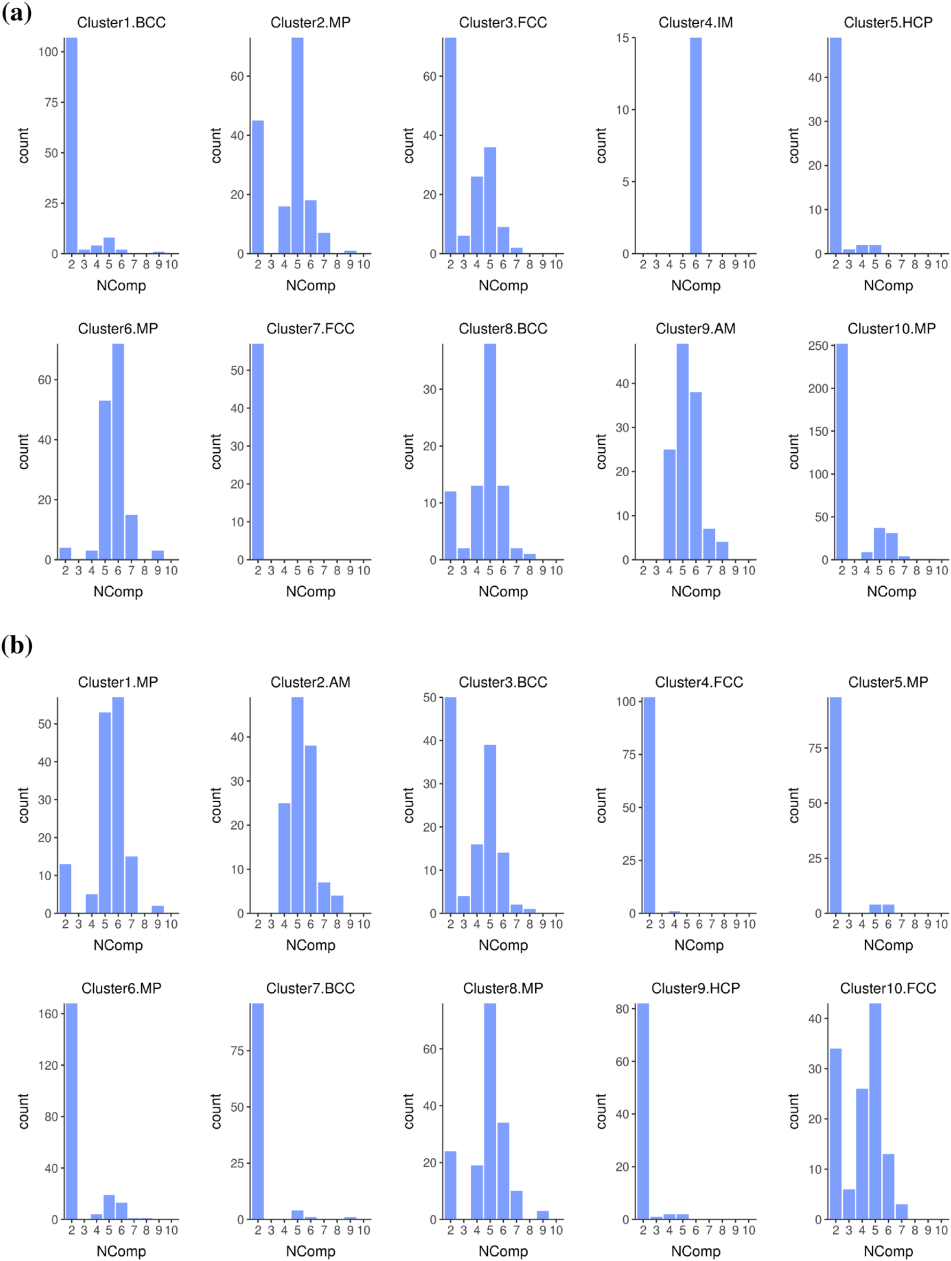
The distribution of the number of components (represented as NComp) for each cluster generated by (**a**) the BD-based and (**b**) SHAP-based data using the *k*-means clustering algorithm, respectively.

In this work, we performed the SHAP analysis ( to complement the BD analysis) that will serve as the input for *k*-means clustering algorithm. The detailed steps are shown in Algorithm 1. The output consists of groups of compositions (or clusters) with similar SHAP values. Once the clusters are identified, we then revisit the individual compositions within each cluster and take an average of the calculated SHAP values and CP profiles. The optimal numbers of clusters from the *k*-means clustering algorithm were identified by plotting the total within sum of square versus the number of clusters (Supplementary Figure S1). According to the literature, it is recommended to choose a *k*-value that can offer the most meaningful interpretation for a given ML model^47^. Although the elbow point is not clear, it was approximately located at 10 clusters after examining several *k*-values. To analyze the results of *k*-means clustering, we plotted the frequency of occurrence of the number of components in the alloy composition for each individual cluster in Fig. 3. This analysis helps to exclude some clusters that are representative of the binary alloys (our primary interest lies in the high entropy alloys, which normally comprise of more than four components). For this reason, clusters 1, 2, 3, 8, and 10 are down-selected from the SHAP results. Our previous BD work^37^ focused on clusters that are representative of the BCC, AM and FCC phases. We now compare the patterns recognized from the SHAP and BD clustering analyses to explain the similarities and differences.
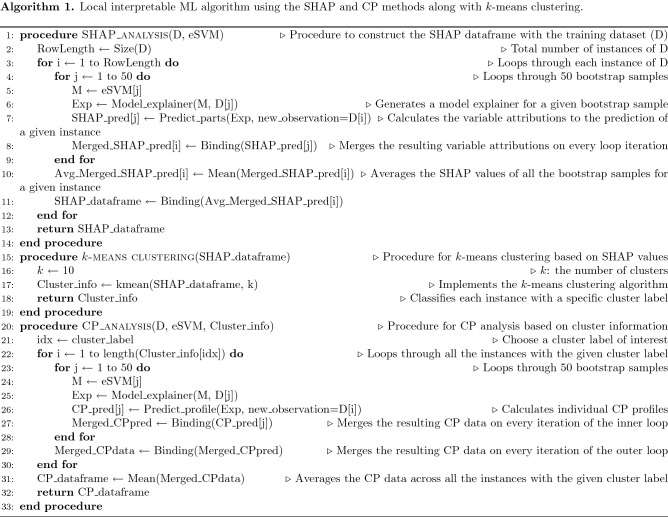

**Figure 4:**
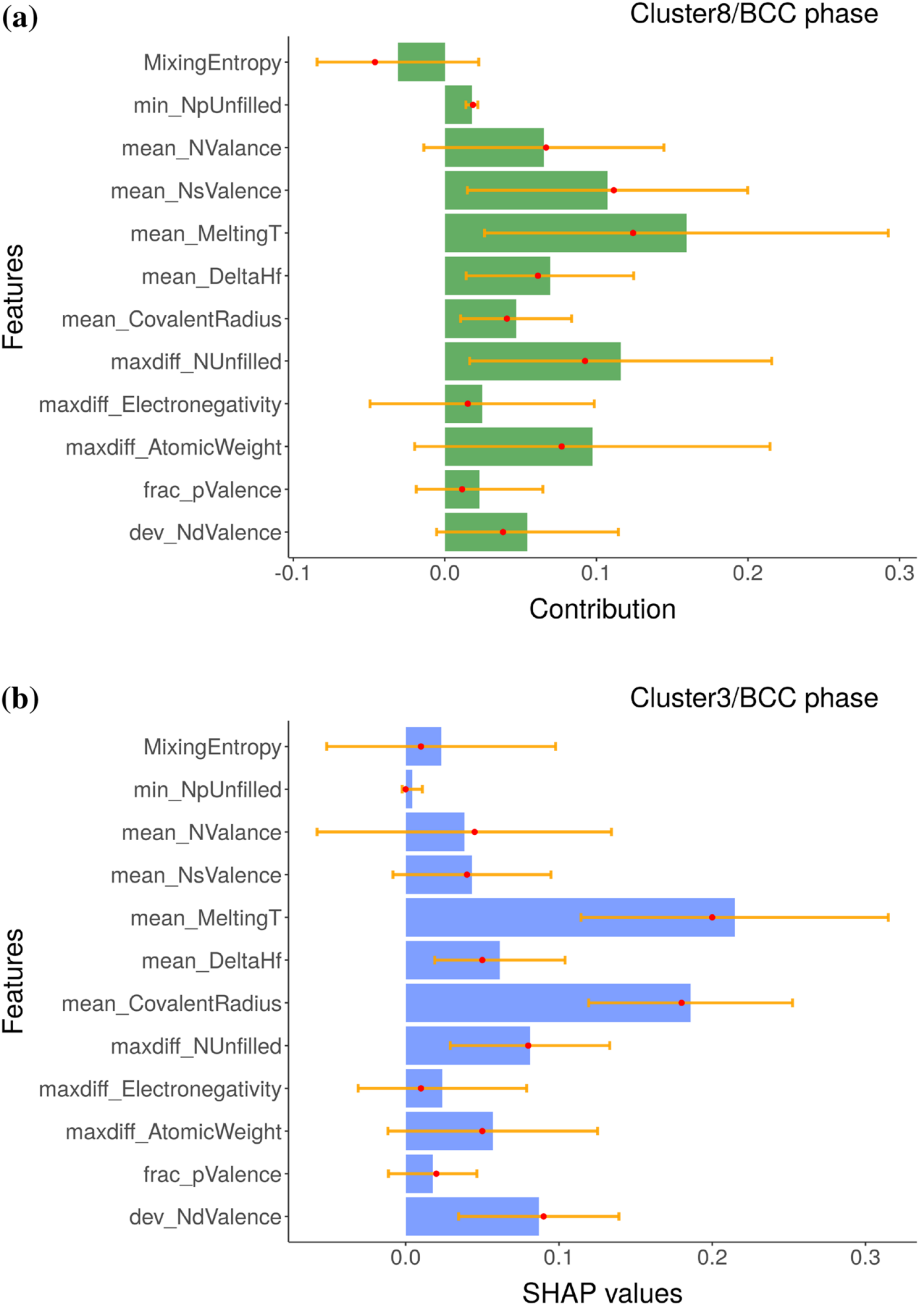
The (**a**) BD and (**b**) SHAP decomposition are plotted for the representative clusters (clusters 8 and 3, respectively), which are predicted to form in the BCC phase by the eSVM model. The size of each bar indicate the averaged contribution of the respective variable towards the overall prediction for a given instance. Red dots and yellow lines denote median values and error bars, respectively. The y-axis of SHAP plot are sorted in the same way as those of BD plot to facilitate the comparison of two plots.

**Figure 5:**
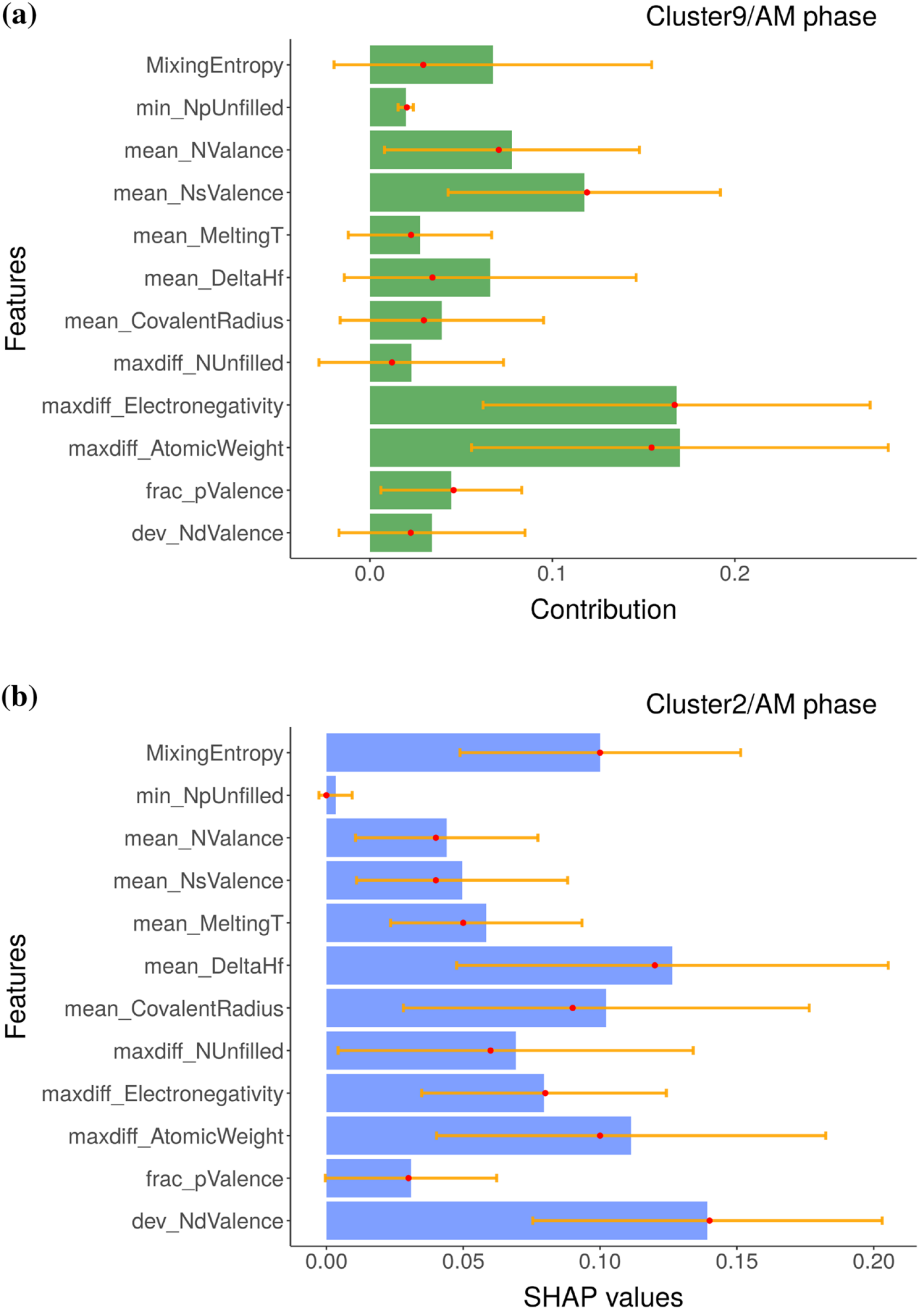
The (**a**) BD and (**b**) SHAP decomposition are plotted for the representative clusters (clusters 9 and 2, respectively), which are predicted to have AM phase by the eSVM model. The size of each bar indicate the averaged contribution of the respective variables towards the overall prediction for a given instance. Red dots and yellow lines denote median values and error bars, respectively. The y-axis of SHAP plot are sorted in the same way as those of BD plot to facilitate the comparison of two plots.

**Figure 6:**
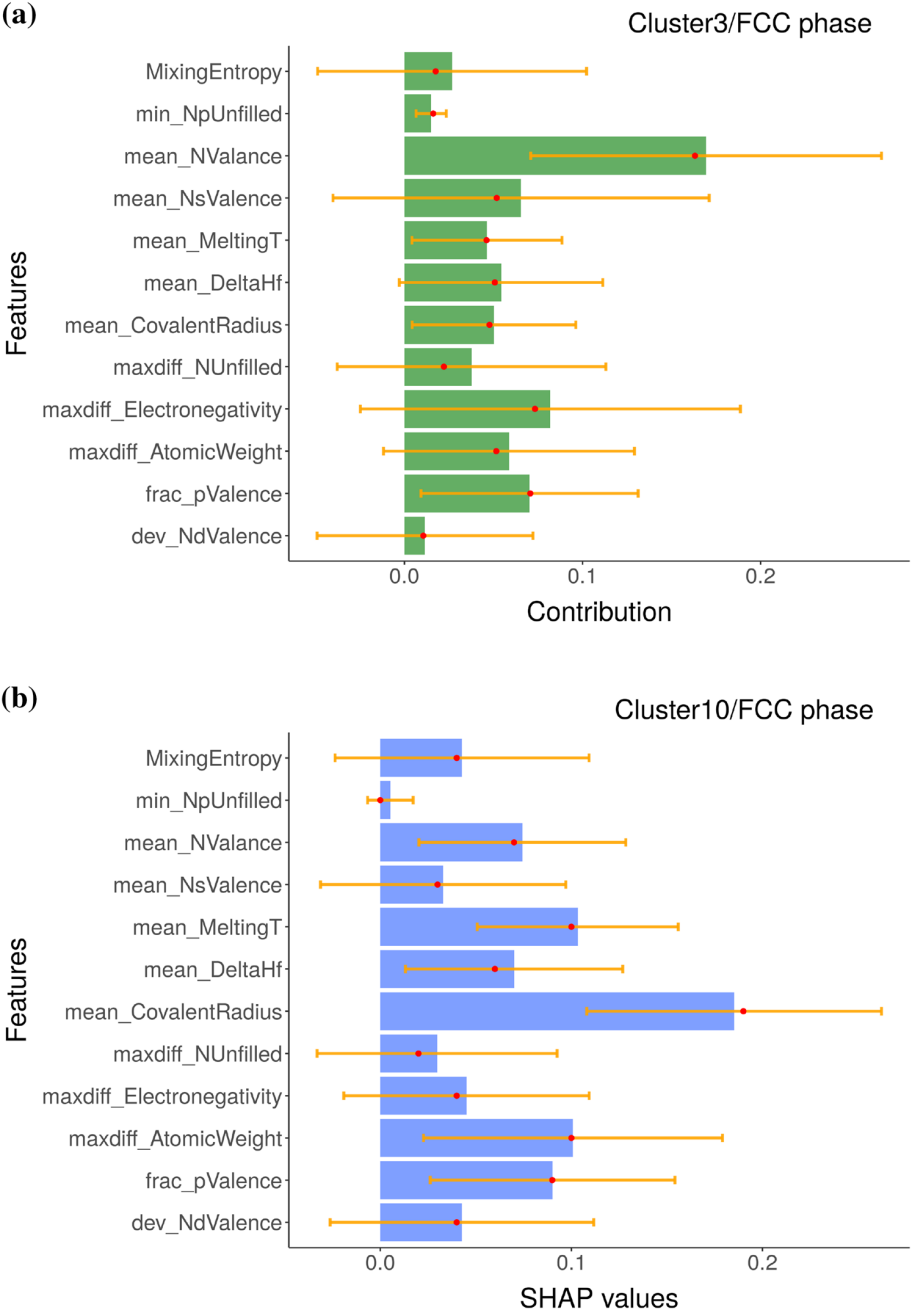
The (**a**) BD and (**b**) SHAP decomposition are plotted for the representative clusters (clusters 3 and 10, respectively), which are predicted to have FCC phase by the eSVM model. The size of each bar indicate the averaged contribution of the respective variables towards the overall prediction for a given instance. Red dots and yellow lines denote median values and error bars, respectively. The y-axis of SHAP plot are sorted in the same way as those of BD plot to facilitate the comparison of two plots.

The local variable attribution analyses based on the *k*-means clustering results are given in Figs. 4, 5, and 6. These clusters can be labeled as containing composition groups that form in BCC, AM, and FCC structures, respectively. Before further interpretation, it is first important to understand the sources of the uncertainty in these plots. We used an ensemble ML approach based on eSVM method for the multiclass classification learning problem. We used the bootstrap resampling method for ensemble learning and we have a total of 50 SVM models in our ensemble. For each of the SVM model, we performed BD, SHAP, and CP profile plots. We then calculated the sample standard deviation, which is visualized as the error bar. Presence of large error bars indicate large model-to-model variation between ensembles, which can be linked to the lack of sufficient number of samples in the training dataset. Given the presence of large error bars, the feature contributions need to be cautiously analyzed. While it is not common in the materials informatics literature to display the feature importance analysis along with error bars, its incorporation offers a better insight into the state of the trained eSVM model. With respect to the BCC phase label (Fig. 4), both the BD and SHAP methods consistently identify mean_MeltingT as the dominant variable. In Fig. 5a and b, we compare the chemical compositions that are grouped in the AM cluster based on BD and SHAP, respectively. Both BD and SHAP methods identify the importance of maxdiff_AtomicWeight variable (albeit maxdiff_AtomicWeight is not the top variable in the SHAP). The BD method also identifies maxdiff_Electronegativity as equally important. The dominant descriptors in the SHAP are the dev_NdValence and mean_DeltaHf variables. Lastly, clustering results that are representative of the MPEA compositions forming in the single-phase FCC structures based on the averaged BD and SHAP data are compared in Fig. 6a and b, respectively. While the BD method identifies mean_NValance as important, the SHAP identifies mean_CovalentRadius as dominant. Both outcomes are consistent with previous published reports in the literature^44,48–53^. Despite exhibiting similar patterns (as visualized in Fig. 2b and c), Fig. 5 and 6 show that the BD and SHAP analyses capture different variable attribution trends to explain the data points that form in the AM and single-phase FCC structures, respectively. These results suggest that there are (likely) different explanations to describe the phase formation rules of MPEAs.

**Figure 7:**
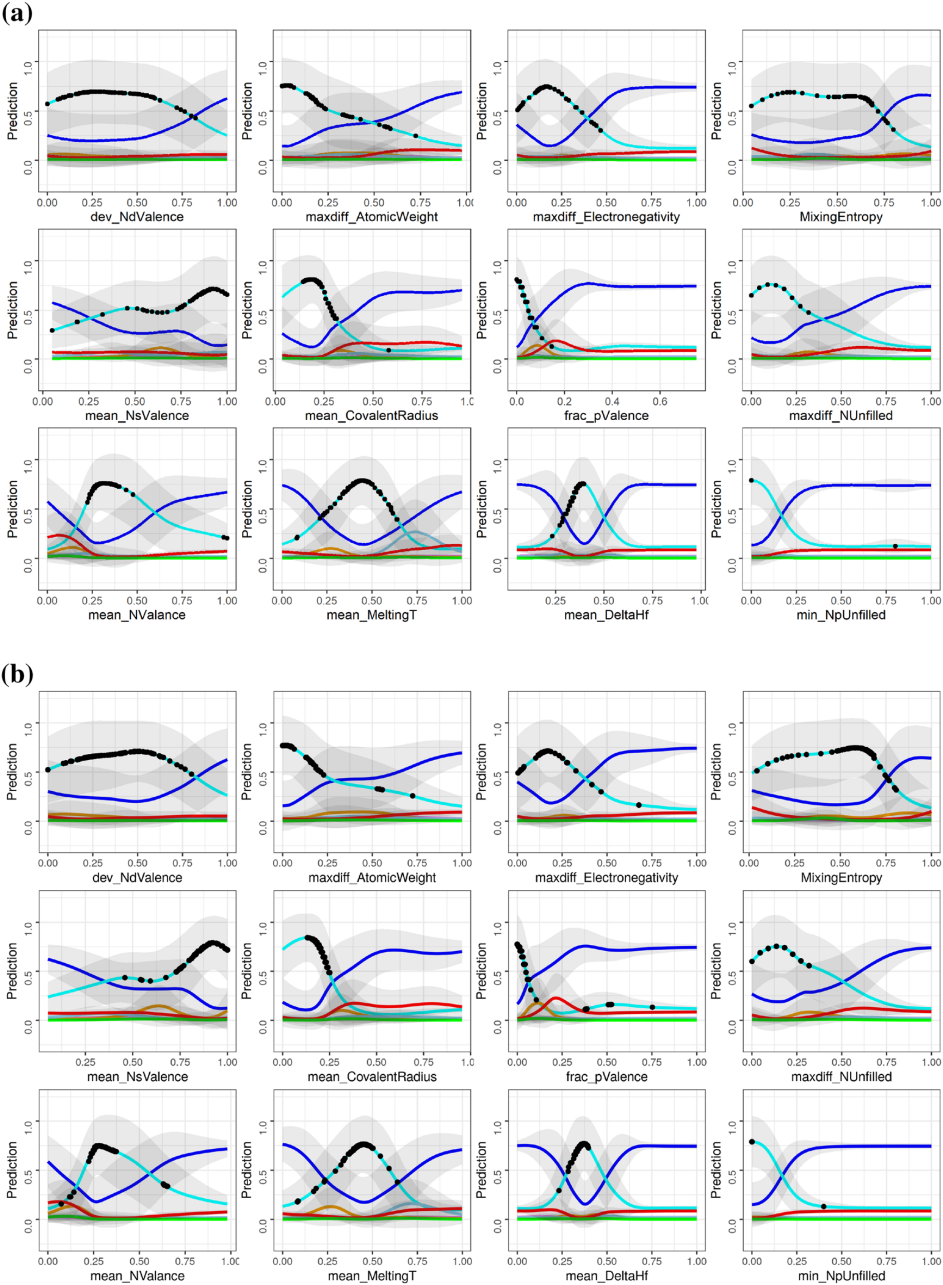
The averaged CP profiles with respect to the 12 input variables based on the (**a**) BD and (**b**) SHAP data of the representative clusters (clusters 3 and 10, respectively), which are predicted to form in the FCC phase by the eSVM model. The black dots indicate the true feature values for all the data points within a given cluster. Line colors denote phase information: blue, MP; violet, AM; cyan, FCC; orange, BCC+FCC; lightblue, HCP; red, BCC; green, IM.

**Figure 8:**
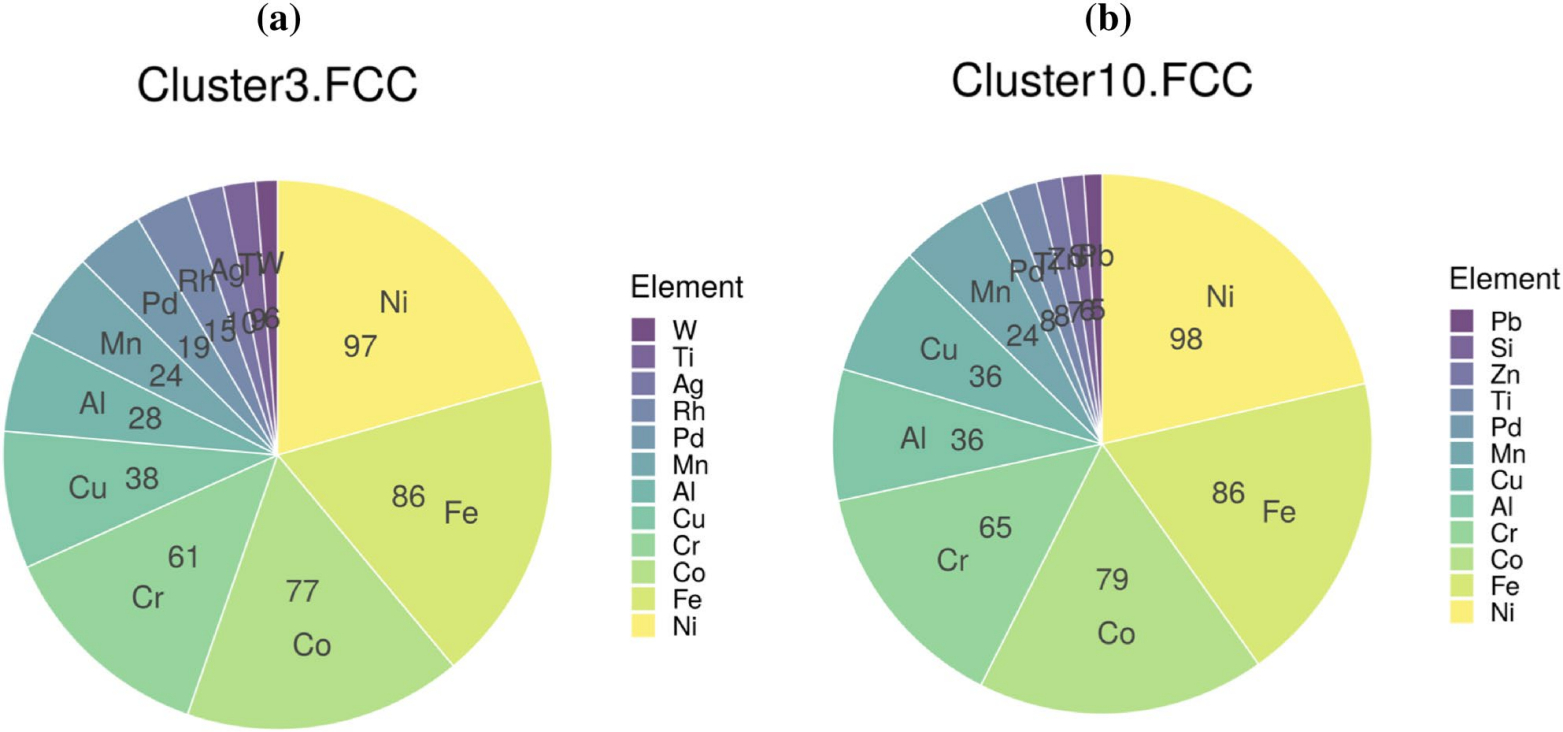
Pie chart showing the distribution of elements in the FCC clusters based on (**a**) BD and (**b**) SHAP decomposition.

The results from clustering analysis of BD and SHAP data are further analyzed through the lens of the averaged CP profile plots. In Fig. 7, we show the averaged CP profile plots for the compositions in the FCC cluster. The black dots in each panel indicate the feature values for each data point within the cluster. There were a total of 152 or 125 compositions that were labeled as FCC in the cluster depending on whether the inputs were from the BD or SHAP data, respectively. When we compared the specific MPEA compositions in the two clusters, we found an overwhelming 87% overlap between them. The pie charts shown in Figs. 8a and b reveal the similar elemental distributions in the two FCC clusters. This is an intriguing result. Despite the differences in the calculation of variable attribution (shown in Figs. 6a and b), we find that similar alloy compositions are grouped in each cluster. This consistency is also reflected in the CP profile plots shown in Figs. 7a and b, where the functional dependencies between each variable and the phases follow a similar pattern. Some minor differences can be found between the BD and SHAP data in the CP profile plots. For example, consider the maxdiff_AtomicWeight variable. In Fig. 7a, the intersection (or the decision boundary) between the cyan (representing FCC phase) and blue (representing presence of Mixed Phases in the microstructure) curves occur at about 0.5 for the normalized maxdiff_AtomicWeight variable. However, the same two curves intersect at $\sim$0.25 for the same variable in Fig. 7b. Nonetheless, the overarching functional dependency looks similar. When we analyzed the averaged CP profile plots for the compositions within the BCC (Supplementary Figure S2) and AM clusters (Supplementary Figure S3), a similar behavior was found. The BCC and AM clusters also show consistent elemental distributions between the two methods (Supplementary Figure S4).

## Discussion

The key discussion points can be summarized as follows. (1) Two-dimensional projection of high-dimensional BD and SHAP datasets based on t-SNE method produced similar data clustering pattern. This result indicates that both BD and SHAP carry information for explaining the phase classification in the MPEA dataset. (2) Variable attribution from BD and SHAP methods do not agree on all levels. There are similarities and differences between BD and SHAP methods. The CP profile plots provide a detailed visual representation of the underlying non-linear relationship between an input variable and the MPEA phase formation probabilities. Both the BD and SHAP methods identify the importance of mean_MeltingT variable for the formation of single-phase BCC MPEAs. The CP profile plots shown in Supplementary Figures S2a and S2b also agrees with this trend, where alloy compositions with high mean melting temperatures are predicted to promote the BCC phase formation. The boxplot shown in Supplementary Figure S5 also captures the uniqueness of the mean_MeltingT variable for the MPEAs forming in the single-phase BCC crystal structure. This result agrees with literature data^54,55^. A similar consistency between the importance variables cannot be reached for compositions that form in the single-phase FCC or AM structures using the BD and SHAP results. We have also developed an interactive web application (https://adaptivedesign.shinyapps.io/AIRHEAD/), where the interested readers can download the BD, SHAP, and CP profile plots for every single alloy composition in the training data. This is in addition to providing a predictive interface for the classification of the expected phase as a function of chemical composition. (3) Despite the substantive difference in the instance-level variable attribution, *k*-means clustering uncovered the common trends between the two methods. We attribute the similarity in the BD and SHAP results to two plausible reasons: (i) Presence of independent and non-interacting input variables. Under this special setting, as Molnar also noted^7^, the differences between the greedy BD method and the exhaustive SHAP method appear to be negligible. (ii) There is more than one unique solution to the local accuracy property of post-hoc model interpretability methods. Thus, we have more than one unique explanation for the phase formation rules of MPEAs. (4) The eSVM method produces large error bars in the calculated variable attribution from both BD and SHAP, which suggests presence of large model-to-model variation between the ensembles. In a recent work, Allen and Tkatchenko^56^ also report the presence of large uncertainties when performing post-hoc model interpretability using the partial dependence plots based on the ensemble kernel ridge regression model. (5) MPEAs that form in single-phase, disordered solid solution are explored as candidate materials for high temperature structural applications^57^. One of the intriguing results we find from the CP profile plots is that there appears to be no direct pathway to transform from single-phase FCC to BCC structures and vice-versa. Figure 7 is a CP profile plot used to visualize the descriptor trends in the MPEA alloy composition cluster that forms in the single-phase FCC structure. In Supplementary Figure S2, we show the CP profile plots representative of the single-phase BCC MPEAs. Intriguingly the blue curve (representative of MPEAs with mixed- or multi-phase microstructures) competes strongly with the cyan curve (single-phase FCC curve in Fig. 7) and red curves (single-phase BCC curve in Supplementary Figure S2). This trend is consistent irrespective of the use of BD or SHAP values in the clustering analysis. The strong competition between mixed-phase and single-phase alloys has been acknowledged before in the experimental MPEA literature^58^.

## Methods

### Dataset, feature selection, and machine learning

The dataset used in this work contains 1,821 compositions, which was created by collecting and preprocessing data from published literature. Each composition is labeled as BCC, FCC, BCC+FCC, hexagonal-closed packed (HCP), Amorphous (AM), Intermetallics (IM), or Mixed-phases (MP) depending on the experimentally determined X-ray diffraction data. A total of 125 input features were added to the dataset using the Magpie program^59^ and down-selected by pair-wise Pearson correlation coefficient (PCC)^60^ within the RSTUDIO environment^61^. Two different thresholds of PCC < 0.4 and PCC < 0.6 were considered for the feature selection step. As there is no standard approach to decide the threshold, we referred to a report from Pei *et al*.^43^ and started from a PCC criterion of 0.6 and then examined a more stricter PCC value of 0.4 to look for further simplification. The correlation analysis resulted in two pre-processed datasets containing 12 and 20 variables for PCC < 0.4 and PCC < 0.6, respectively.

Each dataset was randomly divided into two portions, with 75% for training and 25% for testing. For multi-class classification learning, we constructed the eSVM model where multiple SVM models were generated by the bootstrap resampling method^62,63^ and the nonlinear Gaussian radial basis function kernel was used using the e1071 package^64^. The eSVM hyperparameters were determined using the out-of-bag evaluation based on grid search. The eSVM model performed with a classification accuracy of 86% on the independent test set. The performance metrics are given in Supplementary Table S1. As there was no difference in prediction performance between 12 and 20 feature sets, we selected the simpler 12 feature set which is listed in Table 1 and used for post-hoc local feature analysis to compare SHAP with BD. The details of the dataset and ML model building have been described in our previous paper^37^.

**Table 1:** List of the 12 descriptors selected from 125 descriptors by PCC>0.4.

Notation	Description
maxdiff_NUnfilled	Difference between minimum and maximum numbers of unfilled valence orbitals
min_NpUnfilled	Minimum number of unfilled *p* valence orbitals
maxdiff_AtomicWeight	Difference between minimum and maximum atomic weights
mean_NValance	Average number of filled valence electrons
mean_MeltingT	Average melting temperature
mean_NsValence	Average number of filled *s* valence electrons
dev_NdValence	Standard deviation of the number of filled *d* valence electrons
frac_pValence	Fraction of filled *p* valence electrons
MixingEntropy	Mixing entropy
mean_DeltaHf	Average mixing enthalpy
maxdiff_Electronegativity	Difference between minimum and maximum electronegativity values
mean_CovalentRadius	Average covalent radius of constituent elements

### breakDown, SHAP and Ceteris-Paribus profile plots

Post-hoc model interpretability analysis of black-box models can be broadly classified into two types. One is the global variable importance analysis, where the feature importance is evaluated based on an entire dataset. The other is the local variable importance analysis such as BD and SHAP, where feature importance is evaluated for each individual instance. In this work, we use two of the popular local variable attribution methods: BD and SHAP. The core idea behind BD and SHAP plots is to estimate the relative contribution of an input variable to the model’s prediction by computing the shift in the base or expected value of the response (or the output variable), conditioned on the values of other variables. We adapt the notation scheme of Biecek and Burzykowski^32^ to discuss the differences between the BD and SHAP methods.

In BD, the variable importance measure of the *j*-th variable (or the first *j* variables) for a specific instance ($\underline{x}_{*}$) in the data set can be written as $v(j,\underline{x}_{*})=\Delta ^{(j|J)}(\underline{x}_{*})$, where *j* is an arbitrary input variable, the training dataset has a total of *p* input variables, *J* corresponds to the ordering of *p* input variables as included in the model *f*(), $j \cap J = \emptyset$, $\Delta ^{(j|J)}$ is the change between the expected value of the model’s prediction conditional on setting the first *j* variables (from the set $j \cup J$) equal to their values in $\underline{x}_{*}$ and the expected value conditional on setting the values of the first *J* variables equal to their values in $\underline{x}_{*}$. Thus, the BD analysis explicitly considers the order of the input variables in the model to calculate $v(j,\underline{x}_{*})$. The BD analysis satisfies the property of local accuracy, i.e., $f(\underline{x}_{*})=v_0 + \sum _{j=1}^p v(j,\underline{x}_{*})$, where $v_0$ denote the mean prediction and sum of $v(j,\underline{x}_{*})$ is taken over all *p* input variables such that the $v_0 + \sum _{j=1}^p v(j,\underline{x}_{*})$ is equal to instance prediction from the trained ML model, defined as $f(\underline{x}_{*})$.

In SHAP analysis, the variable importance measure of the *j*-th variable (or the first *j* variables) on an instance $\underline{x}_{*}$ can be written as $\varphi (j,\underline{x}_{*})=\frac{1}{p!} \sum _{J} \Delta ^{[j|\pi (J,j)]}(\underline{x}_{*})$ where the summation is taken over all *p*! permutation or orderings of the input variables. The term $\pi (J,j)$ denotes the set of input variables that are positioned in *J* before the *j*-th variable. Thus, $\varphi (j,\underline{x}_{*})$ can be thought about as the average of the variable-importance measures across all possible orderings of the input variables. Similar to the BD method, the SHAP method is also locally accurate. In addition to local accuracy the SHAP method also carries the following properties: symmetry, dummy feature, and additivity (details can be found in the book written by Biecek and Burzykowski^32^). We can equate $\varphi (j,\underline{x}_{*}) \approx v(j,\underline{x}_{*})$ only in the special case of additive models where the weights associated with each *p* input variable can remain invariant as we change the ordering of the input variables^32^.

While SHAP and BD provide insights about the more general questions such as whether a feature should be included in the model in the first place, or which features are most important to a model’s prediction, CP profile plots provide useful insights into the relationship between each feature and the predicted response for each instance or compositions in our dataset. The CP profile plots are generated by determining the marginal effect of a feature, $\hat{f}(x^{(i)}_{S},x^{(i)}_{C})$, i.e., the change in model prediction as $x^{(i)}_{S}$, the value of a feature under consideration, increases or decreases^32^. In the function described, $x^{(i)}_{C}$ are actual values of other features from the dataset.

The BD, SHAP, and CP methods from the DALEX package^65^ were used for post-hoc local model interpretation, where the contributions of each descriptor to ML prediction were calculated. We performed clustering analysis using the result from BD or SHAP data. We used the *k*-mean clustering algorithm as implemented in the factoextra package^66^. For the local feature importance analysis of each cluster, we averaged the BD or SHAP data of those instances and computed the local feature contributions and CP profiles. In Supplementary Table S2, the cluster numbers from *k*-means clustering are linked to the appropriate phase labels for the ease of interpretation. To visualize the high-dimensional data in two-dimensions, we used the t-distributed stochastic neighbor embedding (t-SNE) method from the Rtsne package^67^ with a perplexity value of 350 and a learning rate of 200.

We also independently analyzed the BD and SHAP data sets using the PCC method to ascertain whether there are any compelling evidences for strong pair-wise linear relationships. The results are visualized as a heat map in Supplementary Figure S6. We find that all pair-wise correlation coefficients in both data sets are less than 0.4, indicating lack of strong pair-wise linear relationships. The correlation coefficient between the BD and SHAP data sets for a common variable is given in Supplementary Table S3. We used three metrics for this purpose: parametric Pearson correlation, non-parametric Kendall rank correlation and non-parametric Spearman correlation^68^. While the Pearson correlation metric measures a linear relationship between the two variables, Kendall and Spearman metrics measure the monotonic relationship. The highest correlation coefficient is estimated to be 0.8 (on the basis of Spearman correlation metric) for the frac_pValence feature. In addition to frac_pValence, min_NpUnfilled and mean_MeltingT were the only other two features whose correlation coefficient exceeded 0.7 with respect to Pearson and Spearman correlation metrics. Other than that, we did not find any evidence for strong correlation between the variables in the two data sets. All variables had a correlation coefficient less than 0.63, when Kendall rank correlation was used. A more detailed analysis can be further performed on the basis of the disentangled representations^69^ to evaluate whether the BD and SHAP data sets have entangled representations. But, this analysis is beyond the scope of this paper and an excellent direction for future work.

## Supplementary Information

Supplementary Information.

## Data Availability

The dataset used for the ML study is freely available in our Web App (https://adaptivedesign.shinyapps.io/AIRHEAD/) and on Figshare^70^.
