# Supplementary material for: A comparison of explainable artificial intelligence methods in the phase classification of multi-principal element alloys
Source: Sci Rep. 2022 Jul 8;12:11591. doi: 10.1038/s41598-022-15618-4 (PMC9270422; doi:10.1038/s41598-022-15618-4)
Supplement: Supplementary file 1 — Supplementary Information. [file 41598_2022_15618_MOESM1_ESM.pdf]

Supplementary Document for

A Comparison of Explainable Artificial Intelligence Methods in the Phase Classification of Multi-Principal Element Alloys

Kyungtae Lee,[1] Mukil V. Ayyasamy,[1] Yangfeng Ji,[2] and Prasanna V. Balachandran[1,3,a)]

[1)] *Department of Materials Science and Engineering, University of Virginia, Charlottesville, VA 22904, USA*

[2)] *Department of Computer Science, University of Virginia, Charlottesville, VA 22904, USA*

[3)] *Department of Mechanical and Aerospace Engineering, University of Virginia, Charlottesville, VA 22904, USA*

(Dated: 16 June 2022)

Algorithm S1. Local interpretable ML algorithm using the BD and CP methods along with *k*-means clustering.

```
procedure BD_ANALYSIS(D, eSVM)    ▷ Procedure to construct the BD dataframe with the training dataset (D)
    RowLength ← Size(D)    ▷ Total number of instances of D
    for i ← 1 to RowLength do    ▷ Loops through each instance of D
        for j ← 1 to 50 do    ▷ Loops through 50 bootstrap samples
            M ← eSVM[j]
            Exp ← Model_explainer(M, D[j])    ▷ Generates a model explainer for a given bootstrap sample
            BDpred[j] ← Predict_parts(Exp, new_observation=D[i])    ▷ Calculates the variable attributions to the prediction of a given instance
            Merged_BDpred[i] ← Binding(BDpred[j])    ▷ Merges the resulting variable attributions on every loop iteration
        end for
        Avg_Merged_BDpred[i] ← Mean(Merged_BDpred[i])    ▷ Averages the BD values of all the bootstrap samples for a given instance
        BD_dataframe ← Binding(Avg_Merged_BDpred[i])
    end for
    return BD_dataframe
end procedure
procedure k-MEANS CLUSTERING(BD_dataframe)    ▷ Procedure for k-means clustering based on BD values
    k ← 10    ▷ k: the number of clusters
    Cluster_info ← kmean(BD_dataframe, k)    ▷ Implements the k-means clustering algorithm
    return Cluster_info    ▷ Classifies each instance with a specific cluster label
end procedure
procedure CP_ANALYSIS(D, eSVM, Cluster_info)    ▷ Procedure for CP analysis based on cluster information
    idx ← cluster_label    ▷ Choose a cluster label of interest
    for i ← 1 to length(Cluster_info[idx]) do    ▷ Loops through all the instances with the given cluster label
        for j ← 1 to 50 do    ▷ Loops through 50 bootstrap samples
            M ← eSVM[j]
            Exp ← Model_explainer(M, D[j])
            CP_pred[j] ← Predict_profile(Exp, new_observation=D[i])    ▷ Calculates individual CP profiles
            Merged_CPpred ← Binding(CP_pred[j])    ▷ Merges the resulting CP data on every iteration of the inner loop
        end for
        Merged_CPdata ← Binding(Merged_CPpred)    ▷ Merges the resulting CP data on every iteration of the outer loop
    end for
    CP_dataframe ← Mean(Merged_CPdata)    ▷ Averages the CP data across all the instances with the given cluster label
    return CP_dataframe
end procedure
```

(a)

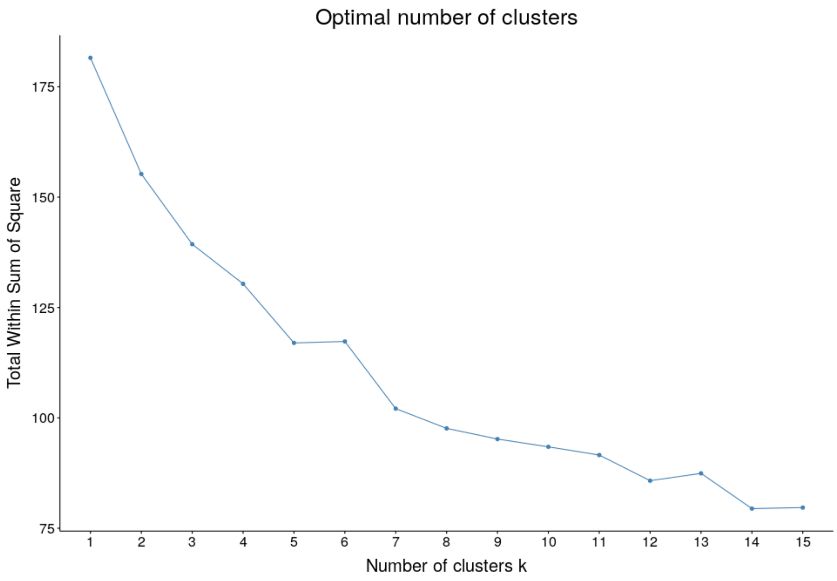

(b)

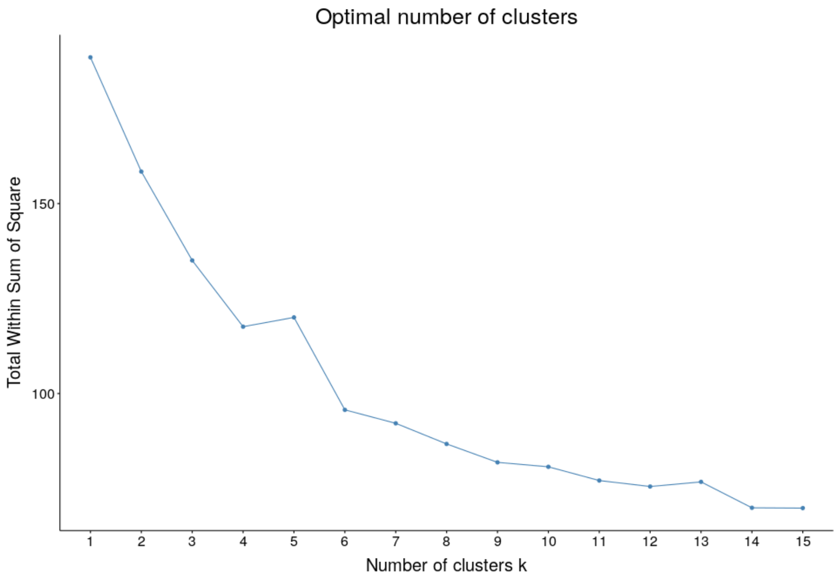

FIG. S1. The total within sum of square is plotted versus the number of cluster by the k-means clustering for the (a) BD and (b) SHAP data.

(a)

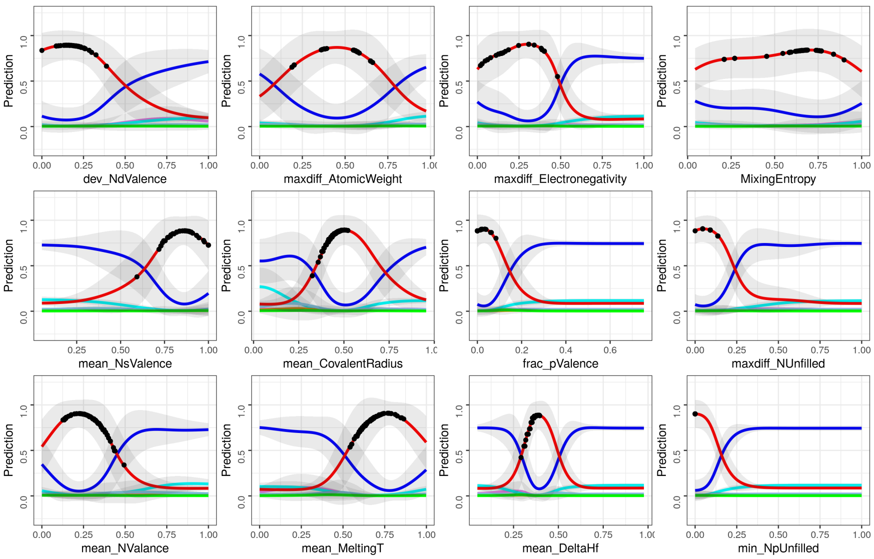

(b)

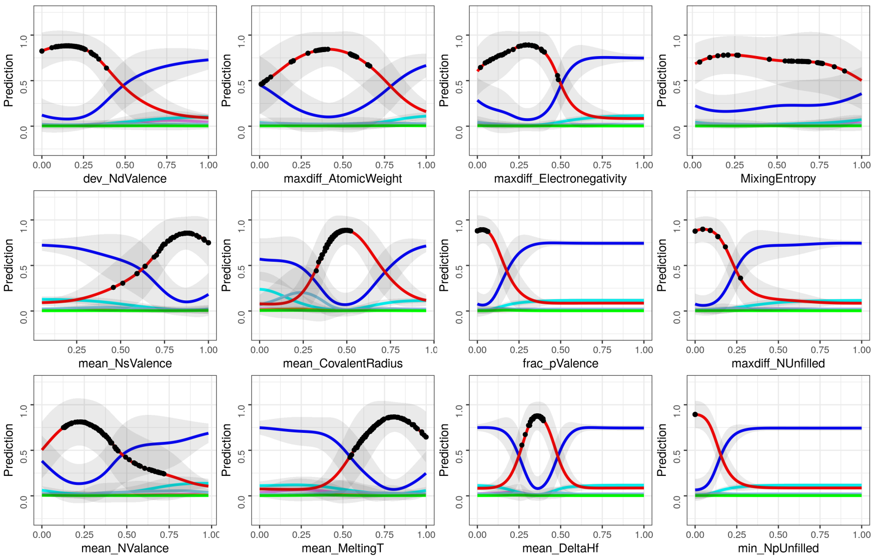

FIG. S2. The averaged CP profiles of the clusters to represent BCC phase with respect to the 12 input variables based on the (a) BD (cluster 8) and (b) SHAP data (cluster 3). The black dots indicate the true feature values for all the data points within that cluster. Line colors denote phase information: blue, MP; violet, AM; cyan, FCC; orange, BCC+FCC; lightblue, HCP; red, BCC; green, IM.

(a)

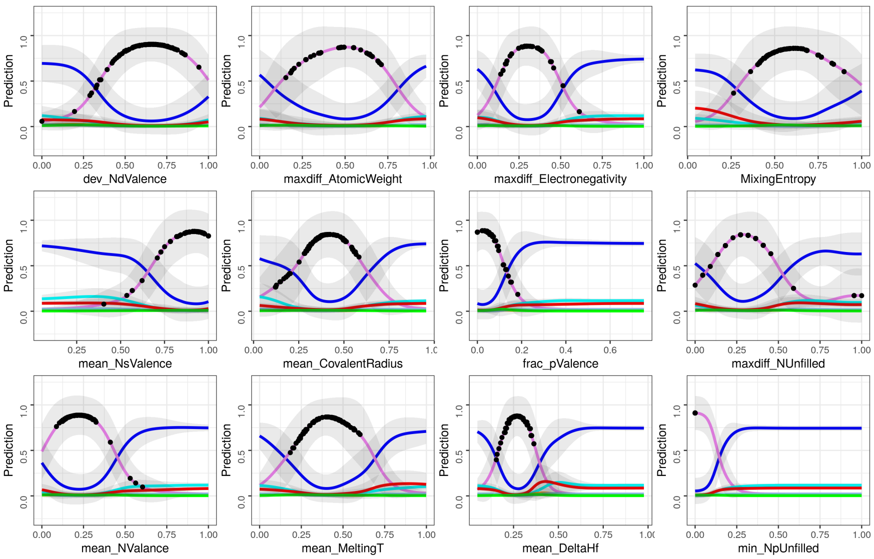

(b)

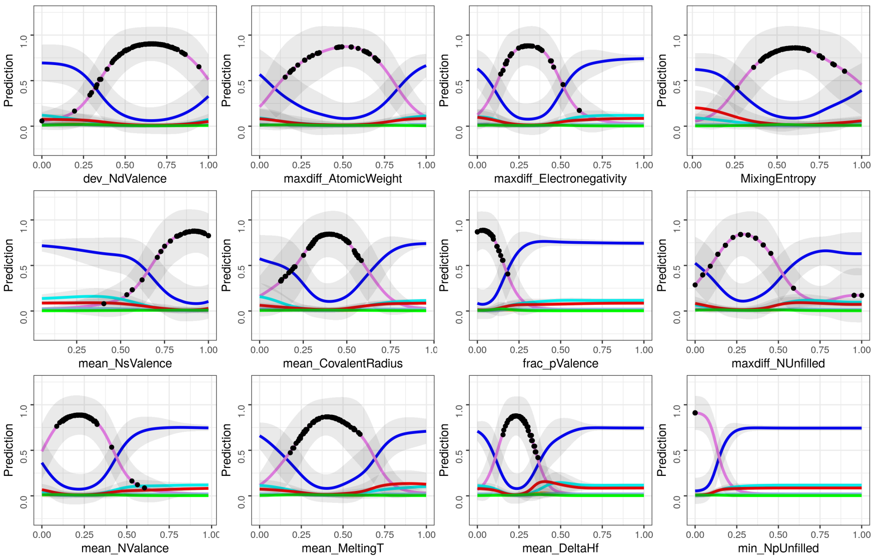

FIG. S3. The averaged CP profiles of the clusters to represent AM phase with respect to the 12 input variables based on the (a) BD (cluster 9) and (b) SHAP data (cluster 2). The black dots indicate the true feature values for all the data points within that cluster. Line colors denote phase information: blue, MP; violet, AM; cyan, FCC; orange, BCC+FCC; lightblue, HCP; red, BCC; green, IM.

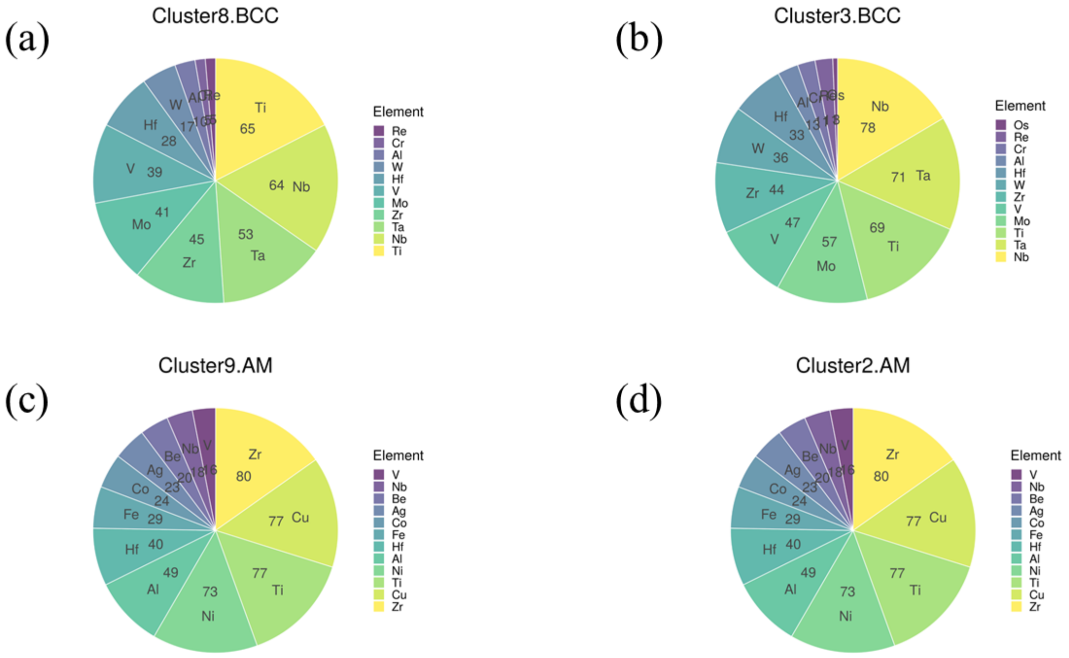

FIG. S4. Pie chart showing the distribution of elements in the BCC and AM clusters based on BD (a, c) and SHAP (b, d) decomposition.

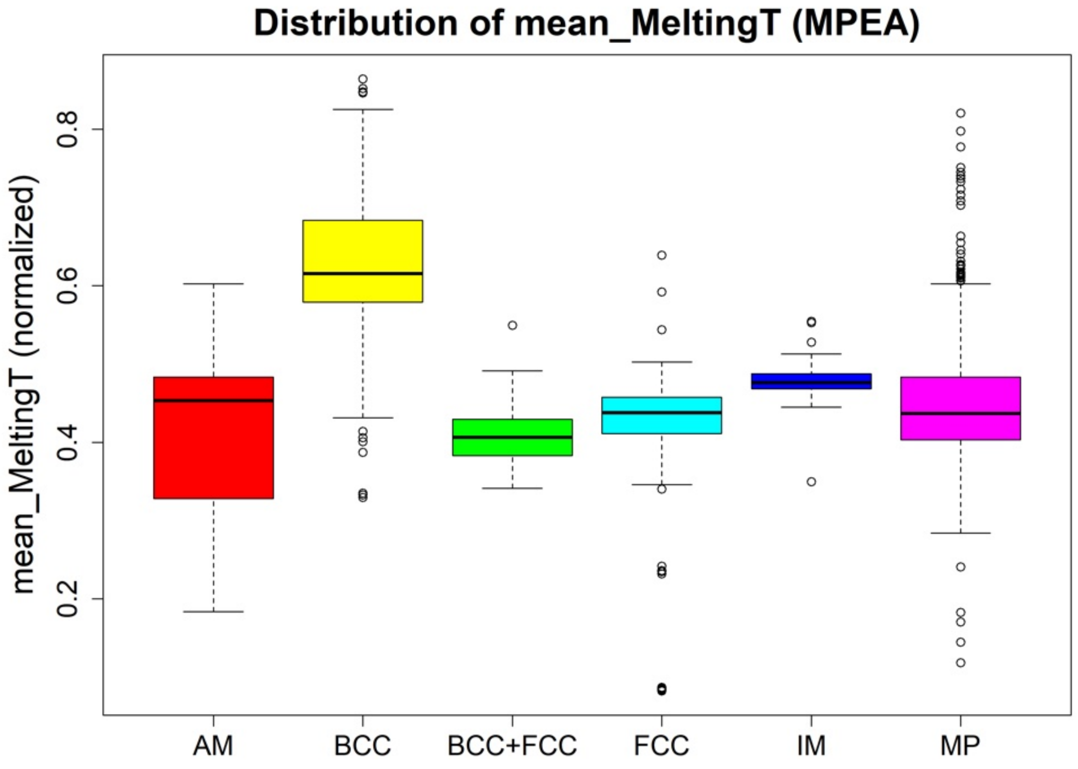

FIG. S5. Distributions of normalized mean_MeltingT input variable with respect to each phase in the dataset where the alloy compositions with more than four elements are only considered.

(a)

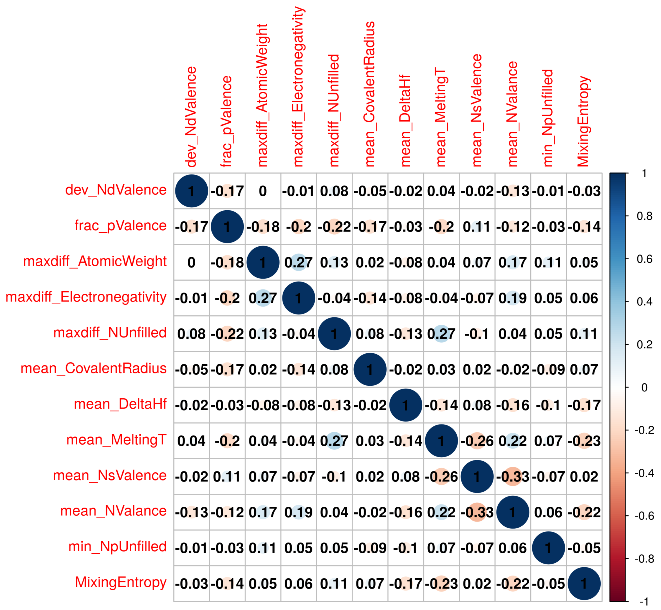

(b)

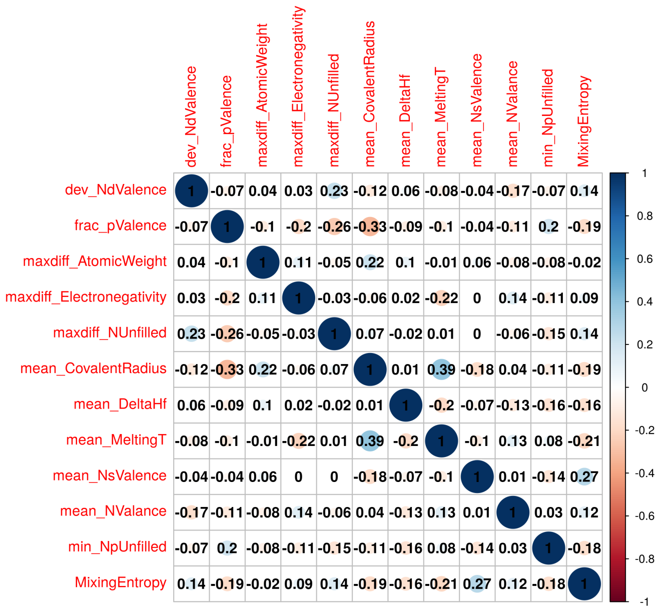

FIG. S6. Pearson correlation matrix heat maps of 12 features that are present in the (a) BD and (b) SHAP data sets, respectively. The size and color of circles describe the pair-wise correlation levels, ranging from -1 (indicating strong inverse or negative correlation) to 1 (indicating strong direct or positive correlation).

TABLE S1. ML predictive performance on the test dataset using eSVM models with 12 and 20 descriptors, denoted as eSVM12 and eSVM20, respectively. Three numbers for each phase indicate the recall, precision, and F1 score of ML prediction, respectively.

	eSVM12	eSVM20
oob error rate	20.1 %	19.1 %
Overall prediction accuracy	85.7 %	87.0 %
AM	0.88, 1.00, 0.94	0.98, 1.00, 0.99
BCC	0.85,0.85, 0.85	0.85, 0.81, 0.83
BCC+FCC	0.53, 0.57, 0.55	0.47, 0.78, 0.58
FCC	0.81, 0.89, 0.85	0.83, 0.88, 0.86
HCP	0.77, 0.85, 0.81	0.87, 0.84, 0.85
IM	1.00, 1.00, 1.00	1.00, 1.00, 1.00
MP	0.91, 0.84, 0.87	0.90, 0.87, 0.88

TABLE S2. The cluster indices are summarized with the corresponding true and BD or SHAP-predicted phases. The phase predictions are carried out by the BD or SHAP methods, followed by the k-means clustering analysis. The true and predicted phases represent the most dominant true and BD or SHAP-predicted phase labels for a given cluster, respectively. The consistency between the true and predicted phases is quantified in the ratio columns where the number of the data points corresponding to a BD or SHAP-predicted phase is divided by the total number of data points for each cluster.

BD prediction				SHAP prediction			
Cluster index (True phase)	Predicted	Ratio	Ratio (%)	Cluster index (True phase)	Predicted	Ratio	Ratio (%)
4 (IM)	IM	15/15	100.0	5 (MP)	MP	106/106	100.00
5 (HCP)	HCP	54/54	100.0	6 (MP)	MP	206/207	99.52
8 (BCC)	BCC	81/82	98.8	3 (BCC)	BCC	126/127	99.21
1 (BCC)	BCC	124/126	98.4	4 (FCC)	FCC	103/104	99.04
9 (AM)	AM	123/126	97.6	9 (HCP)	HCP	87/88	98.86
10 (MP)	MP	333/343	97.1	7 (BCC)	BCC	102/107	95.33
3 (FCC)	FCC	151/161	93.8	10 (FCC)	FCC	124/133	93.23
2 (MP)	MP	145/176	82.4	8 (MP)	MP	161/176	91.48
6 (MP)	MP	129/158	81.7	2 (AM)	AM	123/144	85.42
7 (FCC)	FCC	57/126	45.2	1 (MP)	MP	135/175	77.14
7 (HCP)	HCP	38/126	30.2	2 (IM)	IM	20/144	13.89
7 (BCC)	BCC	26/126	20.7	1 (BCC+FCC)	BCC+FCC	23/175	13.14
6 (BCC+FCC)	MP	13/158	8.2	8 (BCC+FCC)	BCC+FCC	9/176	5.11
2 (BCC+FCC)	MP	13/176	7.4	1 (FCC)	MP	7/175	4.00
2 (FCC	FCC	11/176	6.3	10 (BCC+FCC)	BCC+FCC	5/133	3.76

TABLE S3. Pair-wise correlation coefficient calculated by Pearson, Kendall, and Spearman methods between BD and SHAP data sets with respect to each input feature.

Descriptors	Pearson	Kendall	Spearman
dev_NdValence	0.50	0.33	0.50
frac_pValence	0.77	0.63	0.80
maxdiff_AtomicWeight	0.55	0.38	0.55
maxdiff_Electronegativity	0.56	0.46	0.64
maxdiff_NUnfilled	0.56	0.36	0.53
mean_CovalentRadius	0.55	0.38	0.55
mean_DeltaHf	0.52	0.37	0.53
mean_MeltingT	0.62	0.51	0.71
mean_NsValence	0.38	0.38	0.54
mean_NValance	0.40	0.38	0.55
min_NpUnfilled	0.78	0.50	0.74
MixingEntropy	0.56	0.40	0.57
